# Targeting MFF succinylation: a novel therapeutic strategy for premature ovarian insufficiency by restoring mitochondrial dynamics in granulosa cells

**DOI:** 10.1186/s13048-026-01964-8

**Published:** 2026-01-19

**Authors:** Ying Cao, Xiaoyu Tong, Wei Hu, Yicong Wang, Wenhan Lu, Yuning Chen, Junyi You, Yi Feng, Qingxia Song

**Affiliations:** 1 Department of Gynecology, Suzhou TCM Hospital Affiliated to Nanjing University of Chinese Medicine, 18 Yang Su Road, Gusu District, Suzhou, Jiangsu Province 215009 China; 2 Department of Integrative Medicine and Neurobiology, School of Basic Medical Sciences, Brain Science Collaborative Innovation Center, State Key Laboratory of Medical Neurobiology, Shanghai Medical College, Fudan University, P.O. Box 291, 138 Yi-Xue-Yuan Road, Shanghai, 200032 China; 3 Shanghai Key Laboratory of Acupuncture Mechanism and Acupoint Function, Shanghai Institute of Acupuncture and Moxibustion, Shanghai, 200433 China; 4 Department of Medical Affairs, Suzhou TCM Hospital Affiliated to Nanjing University of Chinese Medicine, 18 Yang Su Road, Gusu District, Suzhou, Jiangsu Province 215009 China; 5 Department of Ophthalmology & Visual Science, Eye & ENT Hospital, Shanghai Medical College, Fudan University, Shanghai, China

**Keywords:** Premature ovarian insufficiency, Mitochondrial fission factor, Succinylation, Post-translational modification, Mitochondrial dynamics

## Abstract

Premature ovarian insufficiency (POI) is a significant clinical disorder characterized by the loss of ovarian function before the age of 40, and its global prevalence is rising. The development of effective therapies is hindered by an incomplete understanding of its pathogenesis. Growing evidence indicates that dysregulated mitochondrial fission in granulosa cells (GCs) is a pivotal contributor to POI, although the upstream regulatory mechanisms remain elusive. This review synthesizes recent findings to propose a novel hypothesis: that aberrant lysine succinylation (Ksucc) of mitochondrial fission factor (MFF) may act as a crucial metabolic switch linking mitochondrial dynamics to ovarian aging. Specifically, hyper-succinylation of MFF at specific residues (e.g., K302) is hypothesized to induce a charge reversal, potentially promoting the excessive recruitment and oligomerization of dynamin-related protein 1 (DRP1) on the mitochondrial membrane. We hypothesize that this leads to mitochondrial fragmentation, bioenergetic deficits, and subsequent apoptosis of GCs and oocytes. This pathogenic cascade is theorized to be driven by a metabolic milieu of elevated succinyl-CoA and diminished desuccinylase activity of SIRT5 in POI. Evidence from related disease models suggests that reversing this imbalance through genetic or pharmacological modulation of SIRT5 can reduce MFF succinylation and restore mitochondrial dynamics. We explore the potential of targeting the SIRT5-MFF axis as a promising therapeutic strategy. Furthermore, detecting elevated MFF succinylation in clinical samples may be explored as a novel diagnostic biomarker for POI, though significant translational hurdles remain.

## Introduction

 Premature ovarian insufficiency (POI), defined as the loss of ovarian function before the age of 40, represents a profound clinical challenge currently estimated to affect 3.7% of women globally [1]. POI not only leads to infertility but also accelerates systemic aging, resulting in long-term health consequences such as osteoporosis, cardiovascular disease, and psychological distress. A particularly devastating aspect for patients is the irreversible decline in the ovarian follicle pool, the functional unit of the ovary. A critical barrier to therapeutic advancement is that over 50% of POI cases are idiopathic, and current mainstay hormone replacement therapy offers incomplete symptom relief without restoring ovarian function or fertility, underscoring the urgent need for mechanism-based strategies.

Granulosa cells (GCs) are indispensable for follicular development and the maintenance of ovarian reserve [2]. Their apoptosis, predominantly mediated through mitochondrial pathways, represents a major mechanism driving follicular atresia and the progression of POI [3, 4]. The ovary, as an organ with high bioenergetic demands, relies heavily on mitochondrial energy supply and functional homeostasis. This dependency intrinsically links mitochondrial abnormalities (e.g., mitochondrial heteroplasmy, dynamics imbalance) to the onset and development of POI [5]. Furthermore, the “mitochondrial dynamics” network, composed of mitochondrial fusion, fission, and mitophagy, serves as a core regulatory mechanism for maintaining mitochondrial functional stability and intracellular homeostasis [6]. Notably, excessive activation of mitochondrial fission disrupts cellular energy metabolism, reducing ATP production and elevating reactive oxygen species (ROS). These metabolic disturbances are strongly correlated with the triggering of the GCs apoptotic program, ultimately accelerating follicle loss and promoting POI progression [7–9].

Despite the established role of aberrant mitochondrial fission in POI, the upstream molecular switches that trigger this process remain elusive. This review explores a novel and emerging regulatory layer: lysine succinylation (Ksucc). We focus on the Ksucc of mitochondrial fission factor (MFF), a key adaptor protein for fission. We critically evaluate the hypothesis that metabolic stress-induced hyper-Ksucc of MFF acts as a critical switch, driving pathological mitochondrial fission, GC dysfunction, and follicular attrition. We examine the current, primarily indirect, evidence supporting this model from ovarian and non-ovarian systems, identify key knowledge gaps, and explore its potential as a therapeutic target for POI.

Ksucc, a metabolite-sensitive post-translational modification (PTM) that transfers a succinyl group to lysine residues, has emerged as a key regulator of mitochondrial proteins since its definitive characterization in 2010 [10]. MFF is a central adaptor that recruits cytosolic dynamin-related protein 1 (DRP1) to the mitochondrial membrane to initiate fission [11, 12]. We hypothesize that pathological Ksucc of MFF acts as a metabolic switch linking mitochondrial dynamics to ovarian aging. Mechanistically, Ksucc of specific lysine residues (e.g., K302) alters MFF’s electrostatic properties, enhances MFF-DRP1 binding, and promotes pathological fission, resulting in bioenergetic dysfunction and apoptosis in GCs [13, 14]. This review explores how Ksucc-induced MFF dysregulation disrupts mitochondrial homeostasis in GCs, underscoring its role in POI pathogenesis, and evaluates the promise of targeting this axis for therapeutic intervention.

## The central role of GC apoptosis induced by mitochondrial dysfunction and dynamics in POI

### GCs as guardians of ovarian reserve

The normal development of ovarian follicles is highly dependent on the function of GCs. As the main somatic cell population in follicles, GCs not only secrete essential factors to support oocyte development but also directly determine follicle fate, with their functional status positively correlated with ovarian reserve capacity [15].

### Mitochondrial pathway of GC apoptosis in POI

Within the follicle, the fate of the oocyte is intimately tied to its surrounding GCs. The viability of GCs is exquisitely sensitive to mitochondrial function, and their apoptosis is a key driver of follicular atresia [16–18], with its core regulation relying on the dynamic balance of mitochondrial fission and fusion. Dysregulation of this network, especially excessive fission, is now recognized as a sentinel event triggering GC apoptosis and POI progression, characterized by reduced ATP synthesis and excessive ROS accumulation, which triggering mitochondrial-dependent apoptotic pathways and accelerating follicular atresia [7]. This is exacerbated by a ROS/mtDNA vicious cycle, wherein excessive ROS induces mitochondrial DNA (mtDNA) mutations through oxidative stress, leading to electron transport chain dysfunction. This, in turn, generates more ROS, forming a self-perpetuating cycle of “excessive ROS → mtDNA damage → further ROS accumulation,” which ultimately exacerbates GC apoptosis and ovarian dysfunction [19–21].

### Critical appraisal of current evidence and unresolved questions

**Fig. 1:**
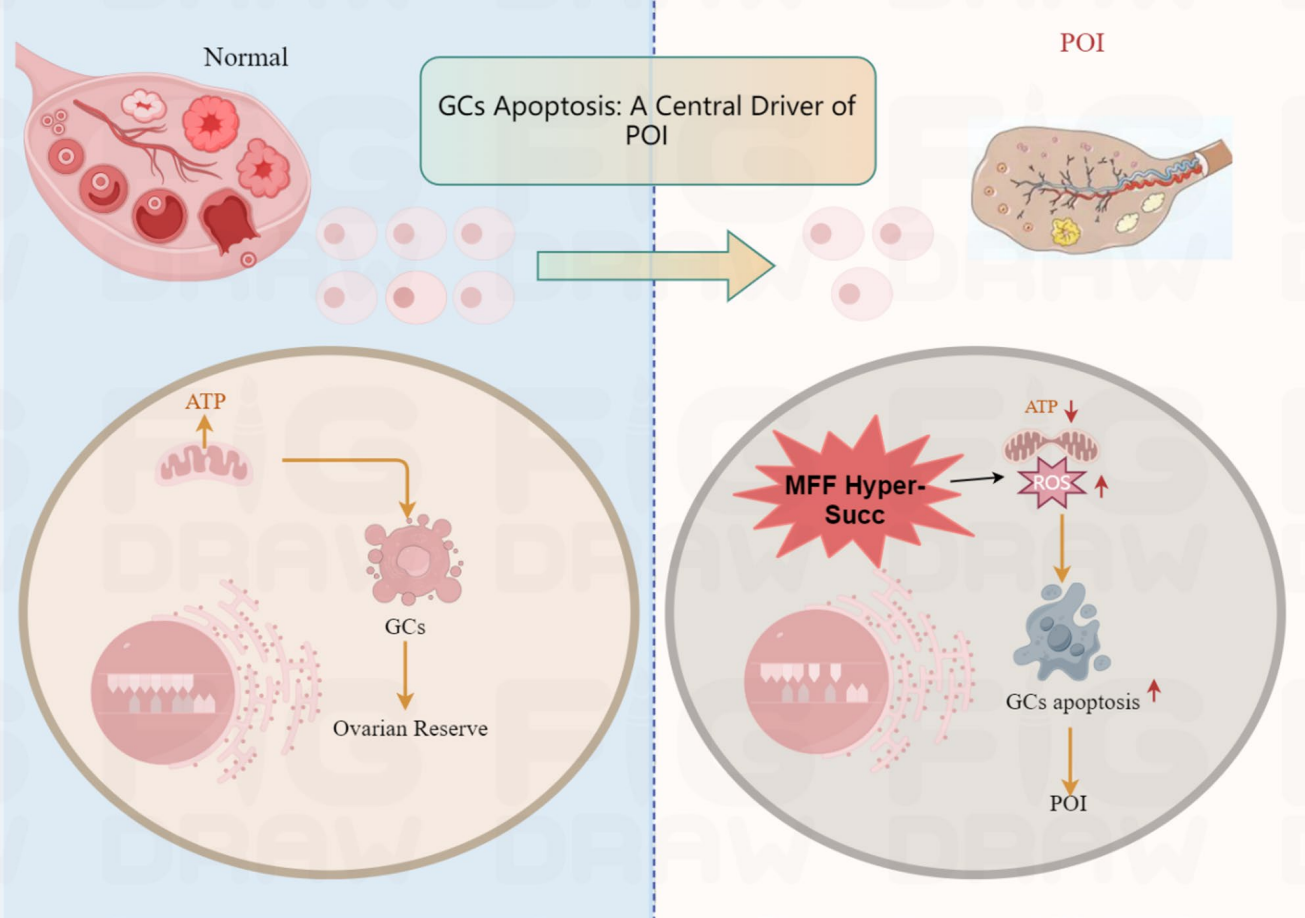
The central role of GC mitochondrial dynamics imbalance in premature ovarian insufficiency (POI). (The left panel illustrates how GCs from healthy individuals maintain ovarian function and reproductive capacity; the right panel demonstrates that excessive or dysregulated GC apoptosis disrupts the follicular microenvironment, accelerates ovarian reserve decline, and leads to POI)

While the correlation between mitochondrial fragmentation and GC apoptosis is strong, several critical questions remain. First, the upstream triggers for the fission/fusion imbalance in POI are not fully defined. Is it a direct response to genomic damage, a consequence of metabolic substrate limitation, or driven by specific signaling pathways? Second, findings on the role of fission can be context-dependent. Some studies suggest transient, regulated fission is necessary for clearing damaged mitochondria via mitophagy, a protective mechanism. The pathological shift in POI may thus represent a failure to resolve fission or to re-initiate fusion, rather than fission per se being universally detrimental [22, 23]. Third, and most importantly for therapeutic targeting, the majority of existing evidence remains correlative. While pharmacological inhibitors of fission (e.g., Mdivi-1) can ameliorate POI phenotypes in some models, these compounds often lack specificity. Definitive establishment of causality requires genetic manipulation (e.g., conditional knockout or knock-in of fission/fusion proteins) in ovarian-specific in vivo models. These knowledge gaps highlight the need to identify precise, druggable molecular switches that convert physiological stress signals into a pathological mitochondrial fragmentation program [24, 25] (Fig.1).

## MFF: a master regulator of mitochondrial fission and GCs fate

### Structure and function

MFF is a tail-anchored protein predominantly localized to the outer mitochondrial membrane (OMM), and serves as a central regulator of mitochondrial dynamics and cellular homeostasis [26]. Its primary function involves acting as a key receptor that actively recruits the dynamin-related GTPase DRP1 to mitochondrial fission sites. MFF directly binds to DRP1, facilitating the assembly of higher-order DRP1 oligomers at mitochondrial constriction sites, thereby driving the mitochondrial membrane fission process essential for determining mitochondrial size and morphology [12]. Notably, the mitochondrial fission process mediated by MFF is not limited to regulating organelle morphology, but is also closely associated with GCs fate: when this fission mechanism is dysregulated, it can ultimately induce GC apoptosis [27]. By specifically targeting and inhibiting the mitochondrial fission process induced by the interaction between MFF and DRP1, it is possible to effectively ameliorate the abnormalities in mitochondrial morphology and functional decline in senescent cells [28, 29]. Thus, MFF acts as an essential upstream factor coupling mitochondrial fission to cell fate decisions and plays an indispensable role in maintaining cellular physiological balance [11, 30].

### Functional coupling to cellular viability

MFF associates with the Voltage-Dependent Anion Channel-1 (VDAC1) at the OMM in vivo. Disruption of this MFF-VDAC1 complex, such as through MFF silencing, triggers a cascade of mitochondrial dysfunction, including increased OMM permeability, loss of inner membrane potential, Ca^2+^ dyshomeostasis, bioenergetic defects, and ultimately, activation of cell death pathways [31]. Notably, MFF downregulation leads to mitochondrial elongation and a concomitant increase in mitochondrial Ca^2+^ uptake capacity, significantly altering cellular function [32].

## MFF hyper-Ksucc: a charge-reversal mechanism disrupting fission fidelity

### Biochemical nature and functional impact of Ksucc

Ksucc is a potent post-translational modification involving the transfer of a succinyl group from succinyl-CoA to lysine ε-amino groups, converting a positively charged side chain (+ 1) into a negatively charged dicarboxylic acid (−1) [33–35]. This significant charge reversal, coupled with increased steric bulk, can profoundly alter protein conformation, interaction networks, and function, serving as a versatile metabolic sensor [36, 37].

### Mitochondrial localization and targets of Ksucc

The mitochondrial matrix represents the epicenter of Ksucc within the cell. This prominence stems directly from its role as the primary site of succinyl-CoA generation-the indispensable substrate for this modification-primarily via the tricarboxylic acid (TCA) cycle enzyme α-ketoglutarate dehydrogenase [38]. Consequently, mitochondrial proteins exhibit exceptionally high levels of Ksucc [39]. This PTM exerts crucial regulatory control over core mitochondrial functions by targeting: ①Metabolic Enzymes: Key enzymes in the TCA cycle (e.g., citrate synthase, malate dehydrogenase) and fatty acid β-oxidation are regulated by Ksucc, impacting flux through these central energy-generating pathways [40]. ②Oxidative Phosphorylation (OXPHOS): Subunits of complexes I, II, Ⅲ, IV, and V (ATP synthase) of the electron transport chain are frequent targets, directly linking Ksucc to cellular energy production efficiency [41]. ③Mitochondrial Dynamics Machinery: Proteins governing mitochondrial fission and fusion, including the central receptor MFF, are subject to this modification, directly linking metabolic state to organelle morphology and quality control.

### Pathological Ksucc of MFF: a hypothesis for impaired fission fidelity

Building on the established role of MFF and Ksucc, we propose a specific hypothesis for POI. MFF constitutes a central regulatory node in mitochondrial quality control, and its function is critically regulated by PTMs. Across diverse pathological contexts—including models of male infertility (e.g., impaired spermatogenesis) [26], metabolic liver disease [13, 14], and ovarian cancer [42, 43]—converging evidence indicates that pathological hyper-succinylation of MFF at conserved residues (e.g., K302) enhances its interaction with DRP1. This modification drives a common pathogenic cascade of excessive mitochondrial fission, which disrupts cellular energy metabolism, exacerbates oxidative stress, and ultimately contributes to tissue dysfunction and disease progression in these distinct systems. This provides a strong mechanistic precedent and forms the basis for our hypothesis that a similar, yet unproven, mechanism operates in POI. We hypothesize that a similar mechanism operates in POI, wherein metabolic stress leads to hyper-Ksucc of MFF. This charge-mediated enhancement could lead to excessive and dysregulated DRP1 recruitment and oligomerization, resulting in pathological mitochondrial fragmentation, bioenergetic collapse, and ultimately, GC apoptosis. The substrate for this modification, succinyl-CoA, is primarily derived from the TCA cycle, and its accumulation under metabolic stress is a key trigger [38, 39]. Preliminary but limited evidence suggests that elevated Ksucc modification in ovarian GCs is associated with mitochondrial dysfunction and increased follicular atresia [44], though direct causality in POI remains to be established.

### SIRT5-mediated regulation of MFF ksucc: translating evidence from bench to ovary

#### SIRT5 as the key desuccinylase regulating MFF ksucc: biochemical basis and extrapolated evidence

The reversibility of Ksucc is enzymatically controlled. The primary mitochondrial NAD⁺-dependent desuccinylase SIRT5 plays a dominant role in reversing Ksucc on mitochondrial proteins like MFF [45, 46]. Foundational evidence supporting SIRT5’s role in regulating mitochondrial fission stems primarily from ovarian and non-ovarian systems. Evidence from cardiomyocytes and hepatocytes have demonstrated that SIRT5 can directly desuccinylate MFF at specific residues (e.g., K302), and that this modification is functionally consequential, regulating DRP1 recruitment and restoring balanced mitochondrial fission [47, 48]. This established biochemical pathway provides a compelling mechanistic precedent for the potential role of the SIRT5-MFF axis in cellular stress responses.

#### Critical appraisal of the SIRT5-MFF axis in the ovarian context

Despite the established role of SIRT5 and MFF Ksucc in other organ systems, their specific functions and causal relationship within ovarian physiology, particularly in POI pathogenesis, necessitate careful and critical examination. A significant translational gap exists: direct evidence demonstrating SIRT5-mediated desuccinylation of MFF in GCs is currently sparse.

Existing studies on SIRT5 in the ovary often report correlative observations, such as altered SIRT5 expression or global shifts in the succinylome in models of aging or ovarian injury [49–51]. However, these studies frequently fall short of establishing: (1) a direct, enzyme-substrate relationship between SIRT5 and MFF within GCs, and (2) the specific functional consequences of MFF (de)succinylation on GC mitochondrial dynamics and survival.

A major interpretive challenge in this field is distinguishing cause from effect. Is a observed decrease in SIRT5 activity or increase in MFF Ksucc a primary, causative driver of mitochondrial dysfunction and GC apoptosis in POI? Or is it merely a secondary epiphenomenon of the broader metabolic collapse and cellular distress? The current literature often cannot resolve this dichotomy. For instance, while SIRT5 deficiency is associated with oocyte meiotic defects [47, 51, 52], it remains ambiguous whether this phenotype is specifically attributable to MFF hyper-succinylation or to the dysregulation of a broader suite of SIRT5 target enzymes involved in core metabolism.

### MFF Ksucc as a proposed convergent pathogenic mechanism in POI

Based on the synthesis of evidence from related fields, we propose a novel pathogenic model for POI, in which aberrant Ksucc of MFF may act as a convergent mechanism disrupting mitochondrial fission and oocyte function [53, 54].

#### Evaluation of evidence linking MFF Ksucc to POI phenotypes

The hypothesis that MFF hyper-Ksucc drives POI pathogenesis is primarily supported by extrapolation from studies in other pathological contexts. For instance, proteomic and functional analyses in oncogenic models have demonstrated that Ksucc of specific MFF lysine residues (e.g., K302) enhances its interaction with DRP1, promoting excessive mitochondrial fission and cellular dysfunction [43]. While these findings establish a compelling precedent, direct evidence within POI is limited and requires rigorous validation.

Corroborating this potential link, mass spectrometry-based proteomics has revealed a global increase in protein Ksucc within ovarian tissues or GCs from certain POI models [44, 55]. These observations are correlated with hallmark POI phenotypes. In vitro studies further suggest that elevated MFF Ksucc is associated with excessive mitochondrial fragmentation and oxidative stress in GC models. However, a critical appraisal reveals major gaps: (1) Most evidence is correlative. (2) Studies specifically demonstrating site-specific MFF Ksucc in validated POI models are lacking. (3) The functional consequence of MFF Ksucc in ovarian cells has not been definitively proven through gain-of-function or loss-of-function experiments. Therefore, future research must prioritize establishing causality, for example, by testing whether genetic or pharmacological inhibition of MFF Ksucc rescues mitochondrial dynamics and ovarian function in vivo [56].

#### Proposed molecular consequences: a charge-driven pathogenic gain-of-function

The postulated pathological impact of MFF hyper-Ksucc is largely attributed to a dramatic charge reversal on specific lysine residues. Based on the established role of electrostatic interactions in protein binding [57, 58], we theorize that this charge reversal alters the electrostatic landscape of MFF’s functional domains, potentially leading to abnormally enhanced and sustained binding to DRP1. This could disrupt the precise spatiotemporal control of DRP1 recruitment and oligomerization, resulting in uncontrolled, excessive mitochondrial fission. This model, while mechanistically plausible, requires direct experimental validation through structural biology and site-directed mutagenesis in ovarian cell systems.

##### Abnormal DRP1 recruitment and oligomerization

Disrupting the charge-dependent binding interface between MFF and dynamin-related DRP1 is hypothesized to enhances MFF’s ability to promote the assembly of higher-order DRP1 oligomers at nascent fission sites-a step essential for mitochondrial constriction [59, 60].

##### Perturbed partner interactions

Ksucc could interfere with mff’s association with regulatory proteins like VDAC1, further dysregulating the fission machinery [55]. The net consequence is a severe impairment of mitochondrial fission homeostasis. This manifests as two interconnected pathologies: (i) prominent morphological changes in the mitochondrial network, typically hyper-fragmentation, and (ii) a profound compromise of core mitochondrial functions, including disrupted Ca²⁺ buffering capacity and impaired bioenergetic metabolism [43]

#### Proposed metabolic triggers and downstream pathogenic cascade

The hyper-Ksucc of MFF is hypothesized to be triggered by a perturbed mitochondrial metabolic environment, such as metabolic stress, elevated succinyl-CoA levels, and/or impaired SIRT5 activity. This dysregulation is proposed to enhance MFF’s ability to recruit DRP1, culminating in excessive fission [61, 62]. The downstream effects are theorized to initiate a vicious cycle: fragmented mitochondria exhibit impaired OXPHOS and elevated ROS; concurrent disruption of Ca^2+^ homeostasis creates a bioenergetic and ionic crisis [63, 64]; this crisis may ultimately trigger apoptosis in GCs and oocytes, driving follicular depletion [65, 66]. This entire cascade, while mechanistically plausible, remains a working model to be tested in the context of POI.

## Therapeutic targeting of the MFF Ksucc axis: from molecular mechanism to translational outlook

Mitochondrial dysfunction characterized by excessive fission and fragmentation in oocytes and GCs has been identified as a key pathological feature of POI [9]. Given the proposed role of MFF Ksucc as an upstream regulator of this process, it emerges as a potential mechanistically grounded therapeutic target [67].

### SIRT5 activation

**Fig. 2:**
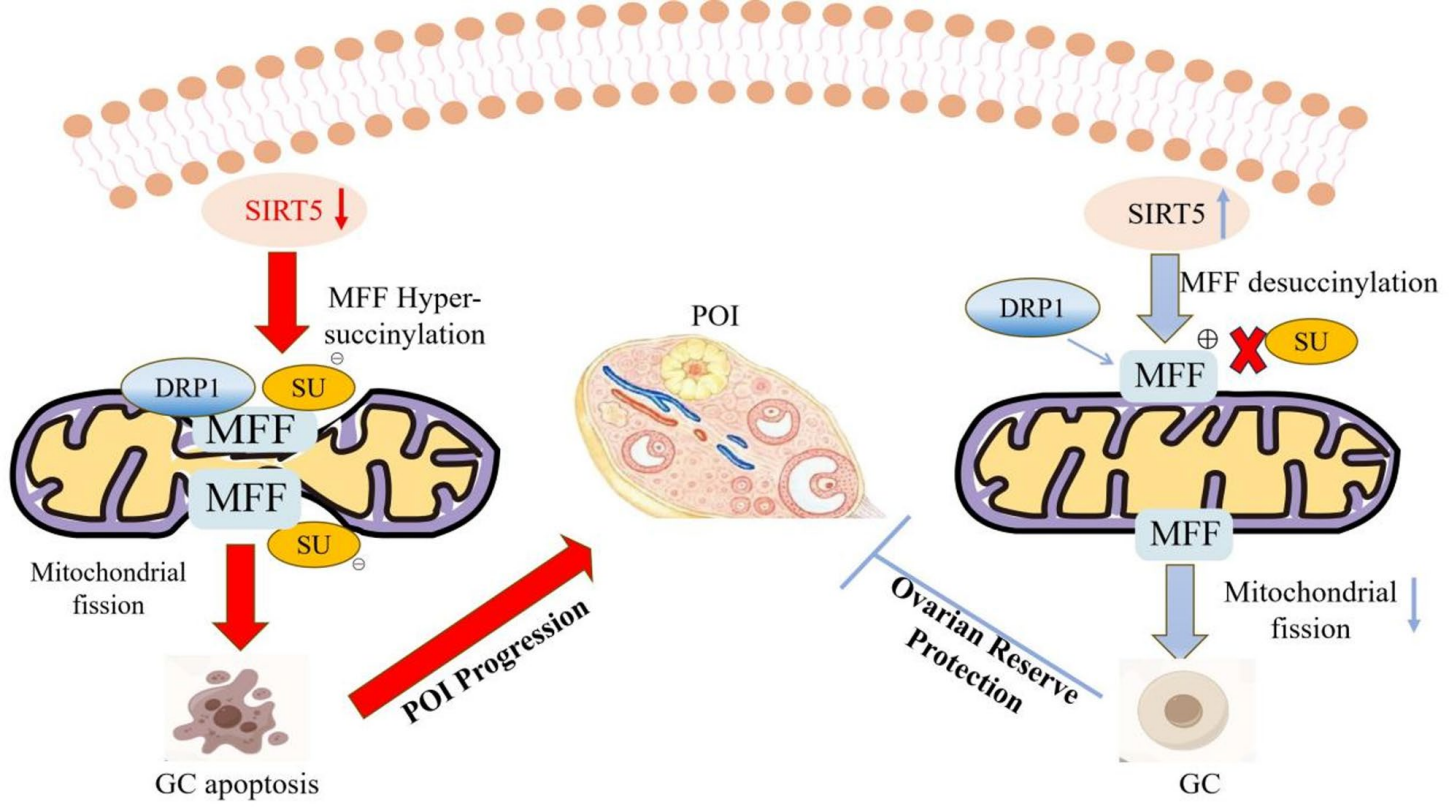
MFF Ksucc: a molecular switch inducing mitochondrial dysfunction in POI and its therapeutic strategies

A primary druggable node with a defined molecular cascade the reversibility of Ksucc makes its regulatory enzymes prime therapeutic targets [68, 69]. SIRT5, the predominant mitochondrial NAD⁺-dependent desuccinylase, stands out as a key mechanistic intervention point [49, 70, 71]. Its therapeutic rationale is grounded in robust genetic and pharmacological evidence, and its mechanism can be delineated as a sequential molecular cascade (Fig. 2):

#### Enzymatic desuccinylation

Activated SIRT5 catalyzes the removal of succinyl groups from specific lysine residues on MFF (e.g., K302). Structural studies indicate SIRT5’s catalytic pocket has a unique preference for recognizing succinylated lysines [72].

#### Conformational restoration and DRP1 recruitment

Desuccinylation restores the native charge and conformation of MFF, enabling proper exposure of its DRP1-binding domain. This facilitates the efficient recruitment of DRP1 to the mitochondrial outer membrane [73].

#### Normalization of fission dynamics

Recruited DRP1 undergoes GTP-dependent oligomerization, executing controlled mitochondrial fission [74]. This corrects the pathological hyper-fragmentation, promoting a balanced, tubular-reticular network.

#### Functional recovery

Mitochondrial morphological normalization directly enhances functional output: it improves OXPHOS complex assembly and efficiency, reduces electron transport chain (ETC) leakage, and attenuates excessive ROS production [75].

#### Ovarian functional outcome

Collectively, this cascade is hypothesized to improve cellular bioenergetics in oocytes and GCs, support key processes like meiotic spindle assembly, and thereby promote follicular survival and development.

### Evaluating the evidence and challenges for SIRT5-targeted therapy

#### Proof-of-concept evidence

##### Genetic evidence

Loss of SIRT5 function in oocytes leads to meiotic defects, including failure of polar body extrusion, cell cycle arrest, and abnormal division, directly linked to elevated Ksucc [49, 50]. Conversely, SIRT5 overexpression reverses hyper-Ksucc and restores normal meiosis and maturation [76, 77].

##### Pharmacological evidence

Small-molecule SIRT5 activators (e.g., compounds like MC3138 or novel derivatives under development) mimic this effect, significantly lowering MFF Ksucc levels [78].

##### Functional outcomes

Both genetic and pharmacological activation of SIRT5 restore DRP1 recruitment and oligomerization, normalize mitochondrial morphology, enhance OXPHOS efficiency, reduce oxidative stress, and improve oocyte quality in vitro and in vivo [79]. Extending these findings to POI requires dedicated preclinical studies in validated POI models.

#### Inherent challenges and limitations

The translation of SIRT5 activation faces significant hurdles:

However, therapeutic activation of SIRT5 presents challenges. As a global mitochondrial desuccinylase, systemic SIRT5 activation could lead to widespread metabolic alterations beyond correcting MFF function, potentially causing off-target effects. The promising evidence above requires dedicated validation in established in vivo POI models. DRP1 recruitment and activity are regulated by multiple adaptors (e.g., MiD49/51) and PTMs, suggesting that targeting SIRT5 alone might be insufficient for a robust therapeutic effect.

### Complementary and alternative targeting strategies

To address the limitations of SIRT5 activation, alternative strategies are under exploration, though they remain at earlier developmental stages:

#### Modulation of Succinyl-CoA levels

Regulating metabolic substrates—such as via anaplerotic agents (e.g., α-ketoglutarate analogs) or inhibitors of succinyl-CoA-producing enzymes may limit substrate availability for Ksucc, mitigating MFF hyper-Ksucc and supporting mitochondrial function [51, 80]. This approach, however, lacks specificity and may disrupt core metabolic pathways, necessitating careful evaluation of the risk-benefit ratio.

#### Site-specific Ksucc inhibitors

Peptides or small molecules designed to block access of succinyltransferases to critical MFF lysine residues could offer high specificity [81, 82], but is currently at a conceptual stage. Rational design would require detailed structural knowledge of the MFF-succinyltransferase interface.

#### Mimetic peptides

Peptides mimicking the non-succinylated DRP1-binding domain of MFF may competitively reduce DRP1 recruitment and fission activity. While mimetic peptides offer theoretical specificity, their clinical translation is hampered by poor membrane permeability and metabolic instability, requiring advanced delivery platforms for effective use.

### Advantages, challenges, and translational considerations

Targeting MFF Ksucc theoretically offers a more precise approach than general fission inhibitors by addressing upstream metabolic dysregulation [83–85]. This strategy not only aims to reestablishes mitochondrial form and function but also may enhances oocyte viability, offering disease-modifying potential for POI [86–89]. However, translating these strategies faces significant challenges: achieving ovarian specificity, ensuring long-term safety of modulating a global PTM, and addressing patient heterogeneity. A critical consideration is functional redundancy; DRP1 can be recruited by other adaptors (e.g., MiD49/51), and its activity is regulated by multiple PTMs (e.g., phosphorylation). Therefore, single-node targeting might be insufficient, suggesting potential utility for dual-target therapies (e.g., combining SIRT5 activation with modulation of other DRP1 regulators). To realize this potential, innovative solutions are needed:

- ①Targeted Delivery Systems: Nanotechnology holds significant promise. Engineered nanoparticles (e.g., PEG-PLGA, liposomes) functionalized with ligands for ovarian follicle cell receptors (ZP3, FSHR) can encapsulate and deliver SIRT5 activators, succinyl-CoA modulators, or anti-Ksucc peptides specifically to ovarian tissue, minimizing systemic exposure and off-target effects [90].
- ②Biomarker-Driven Stratification: Intraovarian injection or implantable sustained-release systems may improve drug bioavailability and efficacy.
- ③Combination Therapies: SIRT5 activators coupled with adjuvants such as antioxidants or mitophagy inducers may synergistically enhance mitochondrial and ovarian function.
- ④Overcoming Translational Hurdles for Biomarker Development: Translating MFF Ksucc into a clinical biomarker is a major hurdle. Profiling Ksucc in circulating extracellular vesicles (EVs) derived from ovarian cells is a promising non-invasive approach [83]. However, significant challenges include: (a) the technical difficulty of isolating ovary-specific EVs from blood; (b) the need for ultra-sensitive detection methods (e.g., targeted mass spectrometry) to quantify low-abundance, site-specific modifications like MFF Ksucc; and (c) establishing clinically relevant thresholds that distinguish pathological from physiological Ksucc levels (Table 1).

**Table 1:** Therapeutic translation: challenges and proposed strategies

Challenge	Proposed Strategy	Potential Impact and Considerations
Off-target effects	Develop ovary-targeted nanoparticles [91] (e.g., ZP3/FSHR ligand-conjugated PEG-PLGA or LNPs) encapsulating SIRT5 activators or Ksucc-blocking peptides	Maximizes ovarian biodistribution, minimizes systemic exposure and toxicity. Requires validation of targeting efficiency and safety.
Functional redundancy	Dual-target therapies [92] (e.g., SIRT5 activator + DRP1-Ser616 phosphorylation enhancer) to synergistically rescue fission	May overcomes potential compensation by alternative fission pathways.Increases combinatorial complexity.
Lack of predictive biomarkers	Profile MFF Ksucc levels in circulating extracellular vesicles (EVs) derived from ovarian cells [93]	Enables non-invasive POI diagnosis, risk stratification, and therapy monitoring. Technically challenging due to low abundance and need for highly sensitive assays.
Patient heterogeneity	Metabolome/succinylome-guided stratification of POI patients [94]	Facilitates personalized therapy selection. Requires establishment of robust omics signatures and clinical correlates.
Chronic treatment delivery	Design long-acting implantable devices (e.g., bioresorbable scaffolds) for sustained intraovarian release [95]	Addresses need for prolonged intervention to preserve follicular. reserve.Involves surgical implantation and biocompatibility issues.

## Conclusion and future perspectives

This review has critically examined the hypothesis that MFF Ksucc serves as a metabolic switch in POI pathogenesis. The pathogenesis and therapeutic strategies of POI remain significant clinical challenges in the field of reproductive medicine. In recent years, mitochondrial dynamics, especially the perpetual cycle of mitochondrial fission and fusion, has been identified as a core regulator of ovarian function, particularly in the somatic cells of follicles [96, 97]. While mitochondrial dysfunction in GCs is established, the upstream regulatory role of specific PTMs like Ksucc is an emerging frontier [98, 99].

We have proposed a model where MFF Ksucc links dysregulated cellular metabolism to follicular attrition [100, 101] and evaluated potential therapeutic strategies targeting this axis [102]. These approaches aim to reestablish mitochondrial integrity, enhance GCs viability, retard follicular depletion, and ultimately offer innovative avenues for clinical intervention in POI.

Despite significant progress, several pivotal questions remain unanswered [44, 103, 104]. Addressing these challenges will deepen our understanding of ovarian aging and accelerate therapeutic development. Key future directions should include:

### Establishing causal evidence in vivo

The highest priority is to generate definitive genetic proof. This necessitates creating knock-in mouse models with GC-specific expression of succinylation-mimetic (K-to-E) and succinylation-resistant (K-to-R) MFF mutations at sites like K302 using CRISPR-Cas9. (The K-to-E (glutamate) mutation mimics the charge reversal of succinylation, testing the “gain-of-function” hypothesis, while the K-to-R (arginine) mutation blocks modification, testing the “loss-of-function/rescue” hypothesis.)These models will directly test if MFF hyper-succinylation is sufficient to induce POI and if blocking it can rescue function, moving beyond correlation to causation [42]. Furthermore, they can define the precise temporal window and cell-type specificity of MFF Ksucc’s role in ovarian aging.

### Developing targeted therapeutics and delivery

The development of highly specific SIRT5 activators remains a major challenge, requiring structure-based drug design. Concurrently, optimizing delivery systems (e.g., FSHR-targeted nanoparticles) is crucial for ovarian specificity and reducing off-target effects [105–107].

### Exploring the ovarian succinylome

Systematic mapping of the succinylome in POI patient samples versus controls using advanced multi-omics is essential [108]. Functional validation using CRISPR/Cas9-based screening and in vitro follicle culture systems would help prioritize the most therapeutically relevant targets [109, 110]. This will reveal whether MFF is a central hub or part of a broader dysregulated network, informing single-target versus network-based therapeutic strategies.

### Translating MFF Ksucc into a clinical biomarker and addressing heterogeneity

Future research should prioritize the establishment of in vitro human follicle culture systems to validate the role of MFF Ksucc in a clinically relevant context. Exploring the succinylome of human oocytes and GCs derived from POI patients versus controls through advanced mass spectrometry will be crucial for identifying patient-specific therapeutic vulnerabilities. The development of ovarian-targeted delivery systems, such as nanoparticles functionalized with Follicle-Stimulating Hormone Receptor (FSHR) ligands, represents a critical step towards clinical translation. Overcoming biomarker translation hurdles requires interdisciplinary collaboration to develop sensitive assays for EV-based Ksucc detection. Future clinical trials must integrate deep phenotyping with metabolomic/succinylomic profiling to identify the “metabolic/PTM-dysregulated POI” subset most likely to respond to SIRT5-targeted therapies [84]. This precision medicine approach is critical for demonstrating therapeutic efficacy.

### Fostering interdisciplinary collaboration

In conclusion, bridging the gap between the proposed mechanism of MFF Ksucc and clinical application in POI will require a concerted, interdisciplinary effort. Cell biologists, structural chemists, bioengineers, and clinical reproductive specialists must collaborate closely. For example, structural chemists and cell biologists could collaborate to determine the crystal structure of succinylated MFF in complex with DRP1, thereby guiding rational drug design. Bioengineers and clinical reproductive specialists can co-develop and validate FSHR-targeted nanoparticle delivery systems. Furthermore, clinical reproductive specialists and bioinformaticians are essential for designing clinical trials that integrate deep phenotypic data with multi-omics profiling from patient cohorts, enabling the validation of biomarker-driven stratification strategies. By rigorously addressing the outlined research priorities and challenges through such integrated teams, we can move closer to validating this novel pathway and potentially developing first-in-class, disease-modifying therapies for POI.

## Data Availability

Not applicable.

## Abbreviations

POI: Premature ovarian insufficiency
GCs: Granulosa cells
MFF: Mitochondrial fission factor
DRP1: Dynamin-related protein 1
TCA: Tricarboxylic acid
Ksucc: Lysine succinylation
PTM: Post-translational modification
mtDNA: Mitochondrial DNA
OMM: Outer mitochondrial membrane
VDAC1: Voltage-Dependent Anion Channel-1
OXPHOS: Oxidative Phosphorylation
EVs: Extracellular vesicles
ROS: Reactive oxygen species
